# Characterization of a Highly pH Stable Chi-Class Glutathione S-Transferase from *Synechocystis* PCC 6803

**DOI:** 10.1371/journal.pone.0126811

**Published:** 2015-05-12

**Authors:** Tripti Pandey, Sudhir Kumar Singh, Gaurav Chhetri, Timir Tripathi, Arvind Kumar Singh

**Affiliations:** Molecular and Structural Biophysics Laboratory, Department of Biochemistry, North-Eastern Hill University, Shillong, India; Utah State University, UNITED STATES

## Abstract

Glutathione S-transferases (GSTs) are multifunctional enzymes present in virtually all organisms. Besides having an essential role in cellular detoxification, they also perform various other functions, including responses in stress conditions and signaling. GSTs are highly studied in plants and animals; however, the knowledge regarding GSTs in cyanobacteria seems rudimentary. In this study, we report the characterization of a highly pH stable GST from the model cyanobacterium- *Synechocystis* PCC 6803. The gene sll0067 was expressed in *Escherichia coli (E*. *coli)*, and the protein was purified to homogeneity. The expressed protein exists as a homo-dimer, which is composed of about 20 kDa subunit. The results of the steady-state enzyme kinetics displayed protein’s glutathione conjugation activity towards its class specific substrate- isothiocyanate, having the maximal activity with phenethyl isothiocyanate. Contrary to the poor catalytic activity and low specificity towards standard GST substrates such as 1-chloro-2,4-dinitrobenzene by bacterial GSTs, PmGST B1-1 from *Proteus mirabilis*, and *E*. *coli* GST, sll0067 has broad substrate degradation capability like most of the mammalian GST. Moreover, we have shown that cyanobacterial GST sll0067 is catalytically efficient compared to the best mammalian enzymes. The structural stability of GST was studied as a function of pH. The fluorescence and CD spectroscopy in combination with size exclusion chromatography showed a highly stable nature of the protein over a broad pH range from 2.0 to 11.0. To the best of our knowledge, this is the first GST with such a wide range of pH related structural stability. Furthermore, the presence of conserved Proline-53, structural motifs such as N-capping box and hydrophobic staple further aid in the stability and proper folding of cyanobacterial GST- sll0067.

## Introduction

Glutathione S-transferases (GSTs; EC 2.5.1.18) are a protein superfamily involved in cellular detoxification [1]. They catalyze the conjugation of a diverse range of electrophilic compounds to reduced glutathione (GSH), thereby playing an important role in the metabolism of xenobiotics including drugs, herbicides and pesticides [1, 2]. The resultant conjugated products are relatively inactive soluble products that can be promptly removed from the cell using efflux pumps [3]. GSTs can detoxify reactive products generated by oxidative stress such as α,β-unstaurated carbonyls, quinines, and hydroperoxides [4]. Isozymes of GSTs are also known to play a role in the biosynthesis of leukotriene C4 and prostaglandins D2, E2 and F2α [5]. Structurally, GSTs are soluble dimeric proteins with each subunit having a molecular mass of approximately 22–28 kDa. The 3D structure of GSTs consists of the same basic protein fold, with each monomer made up of an N-terminal thioredoxin-like domain containing both α-helix and β-sheet and a C-terminal containing all α-helical domains. While the N-terminal domain provides the site for GSH binding, the C-terminal domain contributes most of the amino acid residues that interact with various hydrophobic xenobiotic substrates. The C-terminal domain exhibits more structural variation than the N-terminal domain, presumably to allow the recognition and binding of the structurally diverse range of electrophilic compounds that are known to be GST substrates [1, 6–9]. The subunits of the dimeric enzyme are related by two-fold axis; the N-terminal domain of one subunit interacts with the C-terminal domain of the other [8, 10–15].

GSTs can be divided into at least four major families of proteins, namely cytosolic GSTs, mitochondrial GSTs, microsomal GSTs and bacterial-fosfomycin-resistance proteins [16]. The cytosolic GSTs have been further classified based on several criteria including substrate specificity, immunological cross-reactivity, inhibitor sensitivities, and amino acid sequence similarity [1, 8, 17]. With respect to sequence similarity, GSTs that share greater than 30% identity are assigned to the same class while those with less than 30% similarity are assigned to separate classes [8]. The cytosolic GSTs have been divided into the following classes: mu, alpha, pi, theta, sigma, zeta, and omega. Organism-specific classes of cytosolic GSTs include nu (nematode), lambda, phi and tau (plants), beta (prokaryotes), delta, epsilon, iota and chi (bacteria, insects) [5, 18–23].

GSTs have been widely characterized both structurally and functionally in eukaryotes, where it has been shown to be involved in multiple cellular pathways. In plants, several GSTs have been identified for their roles in oxidative stress tolerance, herbicides, weedicides, and antibiotic resistance [11, 20]. Shishido [24] first reported GST in the bacterium *Escherichia coli* (*E*. *coli*) in 1981. Since then GSTs have been found in a number of prokaryotic organisms. The GSTs found in bacteria belong to beta, chi, zeta and theta classes [21, 23, 25, 26]. The best characterized GSTs in bacteria are PmGST B1-1 from *Proteus mirabilis* and GST from *E*.*coli* [21, 27]. Cyanobacteria constitute a large group of phototrophic bacteria that are widely distributed in nature; they are found in both terrestrial and marine habitats, and some are even extremophiles [28, 29]. The presence of high concentration of GSH in the cytosol of cyanobacteria indicates the presence as well as the importance of enzymes that can utilize GSH in these organisms [30, 31]. These observations suggest important roles of GSTs in cyanobacteria. Recently, Wiktelius et al. [23] described a few properties of *Thermosynechococcus elongatus* BP-1 (TeGST) and *Synechococcus elongatus* PCC 6301 GSTs (SeGST). In order to investigate and characterize the cyanobacterial GSTs, we took a model cyanobacterium *Synechocystis* PCC 6803. Database suggests the presence of at least three GSTs in *Synechocystis*; we initiated our studies using sll0067 GST. On the basis of sequence similarity and catalytic activity, our studies suggest that sll0067 is a Chi-class GST with a high preference for isothiocyanates as substrates. It is highly pH stable and can withstand a pH variation from 2 to 11. To the best of our knowledge, we report the first GST with such an unusual structural stability over a wide range of pH.

## Materials and Methods

### Materials

The molecular biology kits and Ni-NTA agarose were purchased from Qiagen, CA, USA. The dNTPs and enzymes were purchased from New England Biolabs, MA, USA. All other reagents and chemicals were of the highest purity available and were purchased either from Sigma-Aldrich Chemical Company, St. Louis, MO, USA or Sisco Research Laboratories, Mumbai, India. Bacterial culture media was purchased from Himedia Laboratories, Mumbai, India.

### PCR amplification and cloning

The genomic DNA of *Synechocystis* PCC 6803 was isolated and used as a template for polymerase chain reaction (PCR). The GST gene of 0.55 kb encoding for functional GST protein was amplified using gene specific primers (Forward- 5’-CGGGATCCATGATCAAACTATAC-3’ and reverse- 5’-AACTGCAGTCAGCGGGCACC-3’). The PCR conditions used included 98°C for 30 sec followed by 30 cycles (98°C for 10 sec, 66°C for 15 sec, and 72°C for 20 sec), and a final elongation at 72°C for 5 min. The amplified fragments were cloned in the pSK^+^ vector, sequenced and further sub-cloned in pQE30 vector at BamHI and HindIII sites. The resultant constructs were transformed into *E*. *coli* M15 cells for expression.

### Heterologous expression and purification of recombinant sll0067

Recombinant sll0067 was overexpressed in *E*. *coli* M15 cells and purified as follows. A single colony from transformed plates was inoculated in 5 mL Luria Bertini (LB) broth containing 100 μg/mL ampicillin and 50 μg/mL kanamycin. The cells were grown for 12 h at 37°C with continuous shaking at 160 rpm. Subsequently, two 5 mL LB broth tubes containing the above-mentioned antibiotics were inoculated with 1% (v/v) of 4–5 h grown culture and incubated at 37°C with shaking. Cultures were grown until the OD_600_ reached a value of 0.5–0.6; at this stage, the culture was induced with 1 mM isopropyl β-D-1-thiogalactopyranoside (IPTG). The other un-induced culture was used as a control. After 4 h of induction, both the cultures were pelleted by centrifugation at 8000 rpm for 10 min at 4°C. The pellet was then resuspended in lysis buffer that contained a cocktail of protease inhibitors in a total of 1/50 culture volume. The dissolved cells were lysed by sonication, and the lysate was centrifuged at 12,000 rpm for 30 min at 4°C and the supernatant was collected. All further steps were performed under cold conditions. Ni-NTA agarose matrix was equilibrated with equilibration buffer. The supernatant was poured on the affinity column and was allowed to bind slowly. Non-specifically bound, contaminating proteins were removed by washing with equilibration buffer that contained 50 mM imidazole. The recombinant protein was eluted with 10 mL of elution buffer (equilibration buffer containing 400 mM imidazole). The protein was dialyzed against 20 mM potassium phosphate buffer (pH 8.0), containing 150 mM NaCl with or without 2 mM GSH. Protein concentration was determined by Bradford method using bovine serum albumin (BSA) as a standard.

### Size exclusion chromatography

Gel filtration experiments were carried out on a Superdex 200 10/300 GL column (manufacturer's exclusion limit 600 kDa for proteins) on an ÄKTA-FPLC (GE HealthCare Biosciences). The column was equilibrated and run with 20 mM phosphate buffer (pH 8.0), containing 150 mM NaCl and 2 mM GSH with a flow rate of 0.5 mL/min at 25°C.

### Enzyme assay

GST activity using 1-chloro-2, 4-dinitrobenzene (CDNB) and phenethyl isothiocyanate (PITC) substrates was determined spectrophotometrically on the basis of the extinction coefficient for the product S-(2, 4- dinitrophenyl) glutathione (ε_340_ nm = 9.6 mM^-1^cm^-1^) at 340 nm and phenethyldithiocarbamate (ε_274_ nm = 8.89 mM^-1^cm^-1^) at 274 nm, respectively. Here, 1 mL of the assay mixture contained 12 nM enzyme and GSH (1 mM for PITC and 2mM for CDNB) in 50 mM potassium phosphate buffer (pH 6.5 for PITC and pH 8.0 for CDNB), containing 150 mM NaCl. The reaction began with the addition of 0.5 mM CDNB. One unit of GST activity was defined as the conjugation of 1 μmol of the substrate with GSH per minute at 30°C [3]. The data was recorded using a Varian Cary 50 Bio UV-Visible spectrophotometer at 30°C. The pH optimum was determined for CDNB conjugation activity using citrate/glycine/hepes (CGH) buffer of various pH values. Purified sll0067 was incubated at 30°C for 30 min in CGH buffer of pH values ranging from 5.5 to 9.5. Conjugation activity was determined as described above. Three replications were conducted, and the background data were subtracted for all experiments. Kinetic constants were obtained using the Graph Pad Prism software.

### Molecular modeling of sll0067

The dimeric model of sll0067 was generated by a Swiss model based on the template of glutathione transferase SMc00097 from *Sinorhizobium meliloti* with 36.61% sequence identity (PDB ID- 4nhw).

### Secondary structure prediction

Jpred secondary structure prediction was performed using the template of glutathione transferase family member *Xenorhabdus nematophila* (PDB ID- 4l8e).

### Sequence alignments

For similarity analyses, the sequences representing GSTs from Chi class and PmGST B1-1 were aligned using the ESPript 3.0 software [32] that utilizes the Clustal W algorithm [33].

### Fluorescence spectroscopy

Fluorescence emission spectra were measured at 25°C in a Varian Cary Eclipse fluorescence spectrophotometer. In this process, 1 mL of the sample was taken in a 10 mm path-length cuvette and was excited at 280 nm in order to obtain the intrinsic tryptophan fluorescence spectrum. The binding of GSH to the enzyme was monitored by observing the decrease in fluorescence emission intensity of the tryptophan at 330 nm. Both excitation and emission bandwidth was kept at 5 nm. The spectra were collected immediately after adding the GSH. For pH studies, 0.5 μM protein was dissolved in 20 mM CGH buffer of varying pH values (from 2.0 to 11.0) in the presence of 2 mM GSH and was incubated for 2 h at 25°C (pH of the solution maintained) before the fluorescence or circular dichroism (CD) measurements were taken.

### Circular dichroism spectroscopy

Far-UV CD measurements were made with a Jasco J-815 spectropolarimeter calibrated with ammonium(+)-10-camphor sulfonate. The CD spectra were measured at an enzyme concentration of 2 μM with a 1 mm path length cell at 25°C. In a typical experiment, three spectral scans were taken. The values obtained were normalized by subtracting the baseline recorded for the buffer under identical conditions.

## Results

### Purification and oligomeric status of recombinant sll0067

**Fig 1:**
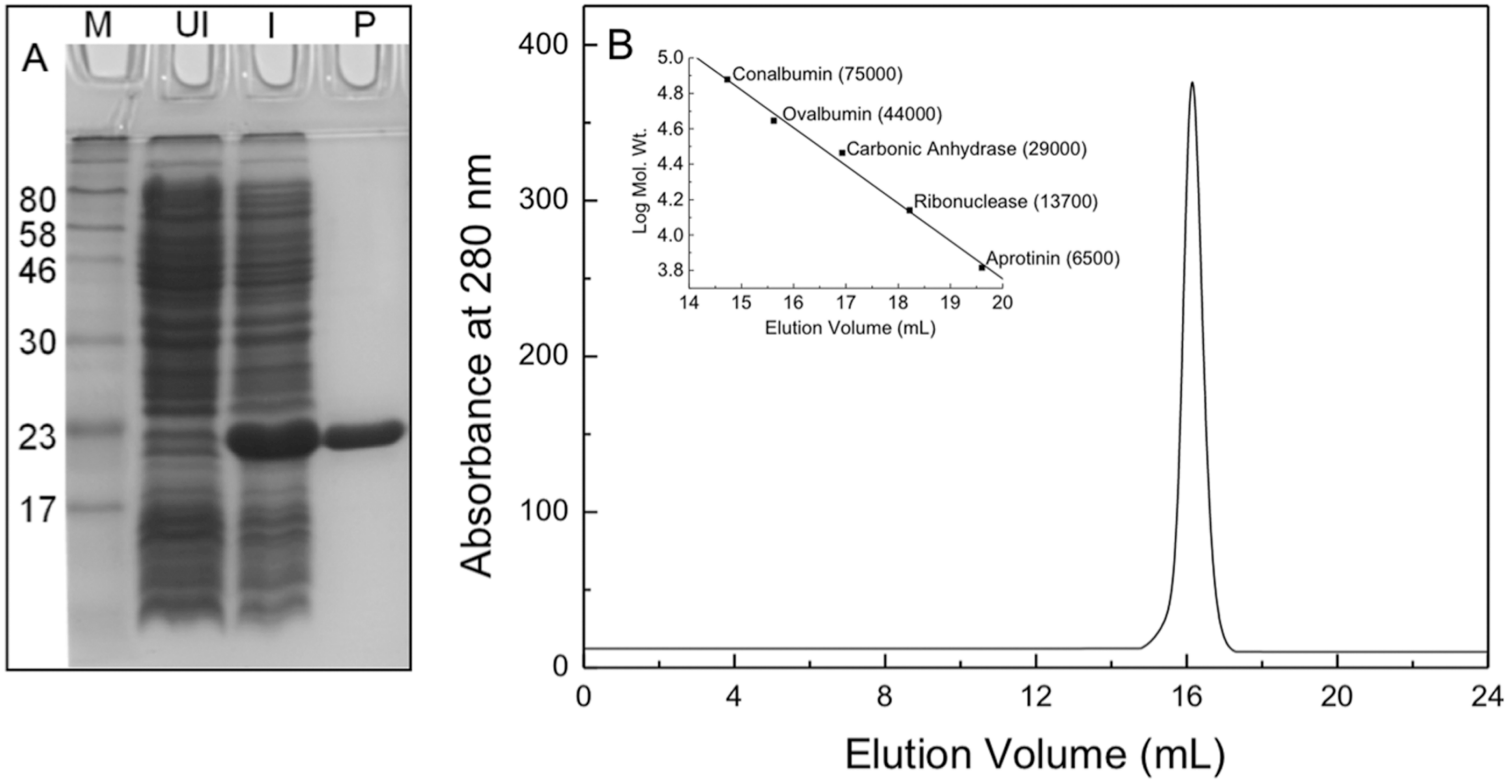
Overexpression of sll0067 in *E*. *coli* and purification of the recombinant protein on Ni-NTA agarose. A. SDS-PAGE analysis of cell lysate showing overexpression of sll0067 and the purified protein. Lanes 1–4 represent molecular weight markers, supernatant of un-induced culture lysate, supernatant of induced culture lysate, and purified protein, respectively. B. Molecular weight and subunit structure of sll0067. SEC profile of sll0067 on Superdex 200 10/300 GL column at pH 8.0 and 25°C. Inset shows the column calibration curve. The column was calibrated with the gel filtration calibration kit containing-conalbumin (75kDa), ovalbumin (44kDa), carbonic anhydrase (29kDa), ribonuclease A (13.7 kDa), and aprotinin (6.5 kDa).

The recombinant plasmid was transformed into *E*. *coli* M15 cells for functional expression. The protein was purified to homogeneity using Ni-NTA agarose matrix. Fig 1A summarizes the expression and purification of the recombinant sll0067. The oligomeric status of the protein was determined using size-exclusion chromatography (SEC). On Superdex S-200 column, the protein eluted at a volume of 16.1 mL that corresponds to about 40 kDa when compared with molecular weight standards, indicating that the protein existed in a dimeric state under non-denaturating conditions (Fig 1B). This was further confirmed using the glutaraldehyde-induced crosslinking that showed a band of about 40 kDa in sodium dodecyl sulfate-polyacrylamide gel electrophoresis (SDS-PAGE) (data not shown).

### Enzyme activity analysis

The purified protein was kinetically examined with CDNB as a model substrate for GST catalyzed reactions. The protein has good GSH-transferase activity with CDNB, i.e. *k*cat and *K*m. However, with its class specific substrate- isothiocyanates, sll0067 showed strong catalytic activity, having *k*cat of 15.15 s^-1^ and high enzymatic affinity with km value of about 0.3193 mM. The kinetic parameters were determined for CDNB and PITC. The specific activities with CDNB and PITC were found to be 4.62 ± 0.68 μmol.min^-1^.mg^-1^ and 43.66 ± 3 μmol.min^-1^.mg^-1^, respectively. The ITC substrates were by far the best substrates tested for sll0067 as compared to CDNB, as the specific activity of sll0067 is approximately 10 times higher in the presence of PITC. The enzyme sll0067 has maximum enzymatic efficiency with PITC. The kinetic parameters with GSH and PITC are given in Table 1.

**Table 1:** Steady state kinetic parameters.

	GSH	PITC
Km (mM)	0.9185 ± 0.18	0.3193 ± 0.033
Vmax (μmol.min^-1^)	12.92 ± 0.075	21.83 ± 0.84
Kcat (sec^-1^)	8.97 ± 0.48	15.15 ± 0.58
Kcat/Km(sec^-1^.mM^-1^)	9.76 ± 0.66	47.47 ± 0.62

Enzymatic activities are measured at various concentrations of GSH and PITC as described in the Experimental section. Kinetic constants are based on three independent experiments for each measurement. Results are mean ± standard deviation (S.D.) of five independent measurements as described in the Experimental Section.

### Effects of pH and temperature on enzymatic activity

**Fig 2:**
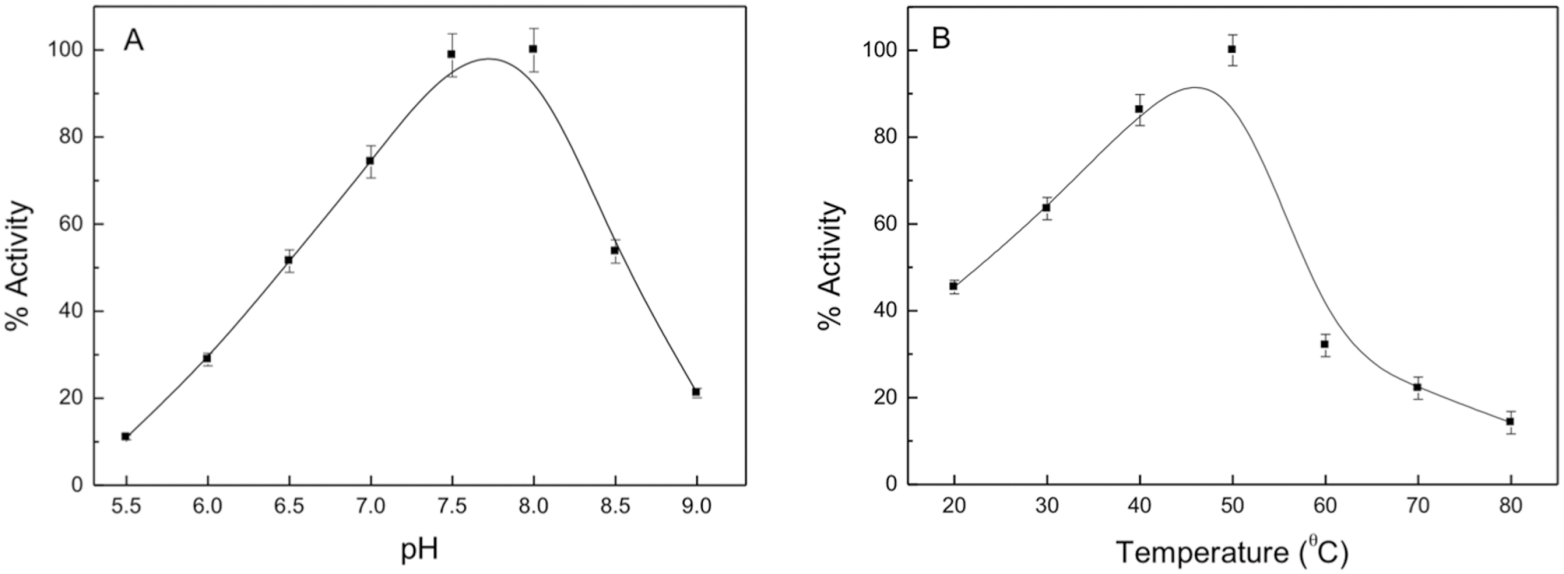
Studies on the effect of pH and temperature of the enzymatic activity of sll0067. A. Effect of pH on the catalytic activity of sll0067. B. Effect of temperature on the activity of sll0067.

The pH optimum of sll0067 with CDNB as substrate was found to be 8.0. At the pH value below 6.5 and above 8.5, the activity decreased substantially (Fig 2A). Temperature dependent studies revealed maximum sll0067 activity at 50°C while the activity was reduced to approximately 40% at 20°C. In addition, the activity decreased significantly at high temperatures, i.e., the activity reduced to 30% at 60°C and 20% at 80°C (Fig 2B).

### Effect of pH on the structural stability of sll0067

**Fig 3:**
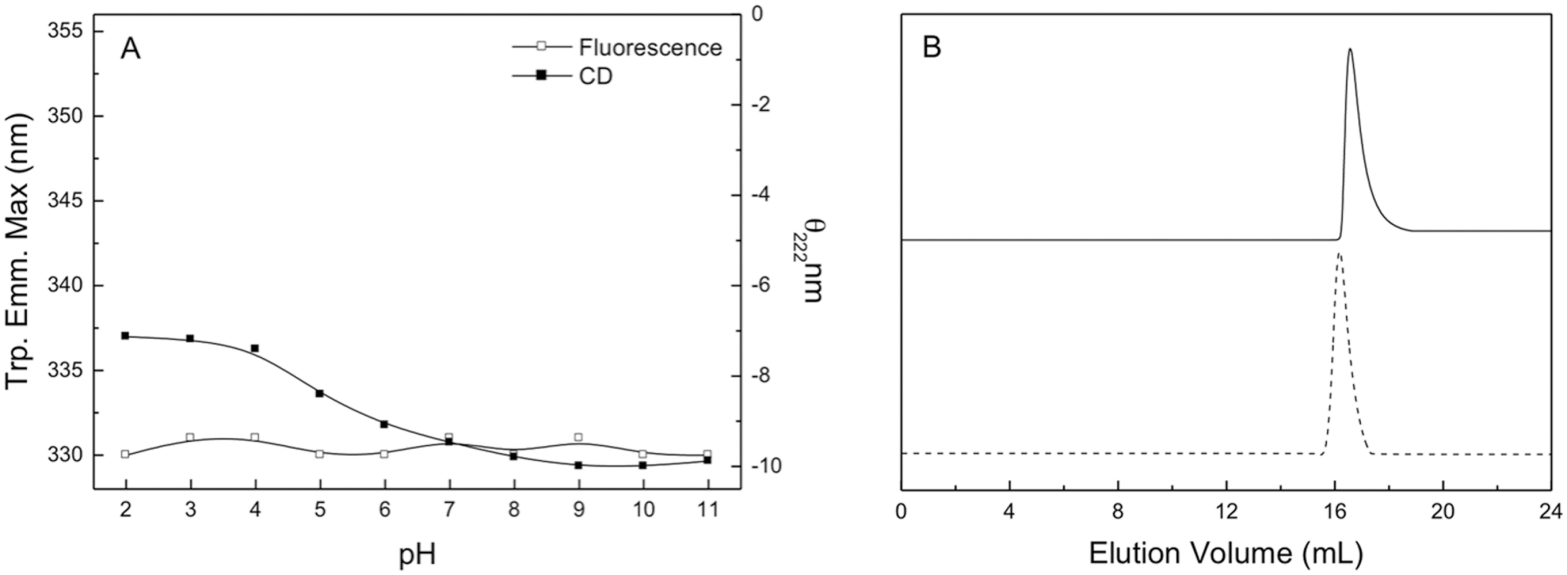
Confirmation of the integrity of the sll0067 structure. A. Effects of the pH on the CD signal at 222 nm (■) and tryptophan fluorescence emission maxima (□) of sll0067. B. SEC profile of sll0067 on Superdex 200 10/300 GL column at pH 3 and 10 at 25°C. Solid lines represent the protein at pH 3 while the dashed lines represent the protein at pH 10. The curves have been displaced on Y-axis for presentation.

In order to study the pH-induced changes in the structural properties of sll0067, the protein was incubated with CGH buffer of different pH values, ranging from 2 to 11. The effect of pH on the secondary, tertiary and quaternary structure of sll0067 was studied using the far-UV CD, fluorescence spectroscopy, and SEC, respectively. Fig 3A summarizes the effects of pH on the CD signal at 222 nm and the intrinsic tryptophan fluorescence of sll0067. No significant changes in the tryptophan fluorescence were observed; however, only minor changes were found in the CD signal during the entire variation in pH, indicating no major alterations in the secondary or tertiary structure of the protein. The minor change in CD signal at very low pH can be due to the unfolding of few local secondary structures. In order to confirm the results of CD and fluorescence studies, pH-dependent SEC was performed. At pH 10, the protein eluted at an elution volume of 16.1 mL that is same as that of the dimeric protein at pH 8.0, whereas at pH 3, a minor shift in the elution volume was observed (16.6 mL). These results indicate that the quaternary structure of the protein was intact at these pH values (Fig 3B). Overall, these results suggest that the complete topological structure of sll0067 was retained between pH values of 2 to 11 and that there is no change in the global architecture of the protein even at extreme pH values.

### Binding of GSH to sll0067

**Fig 4:**
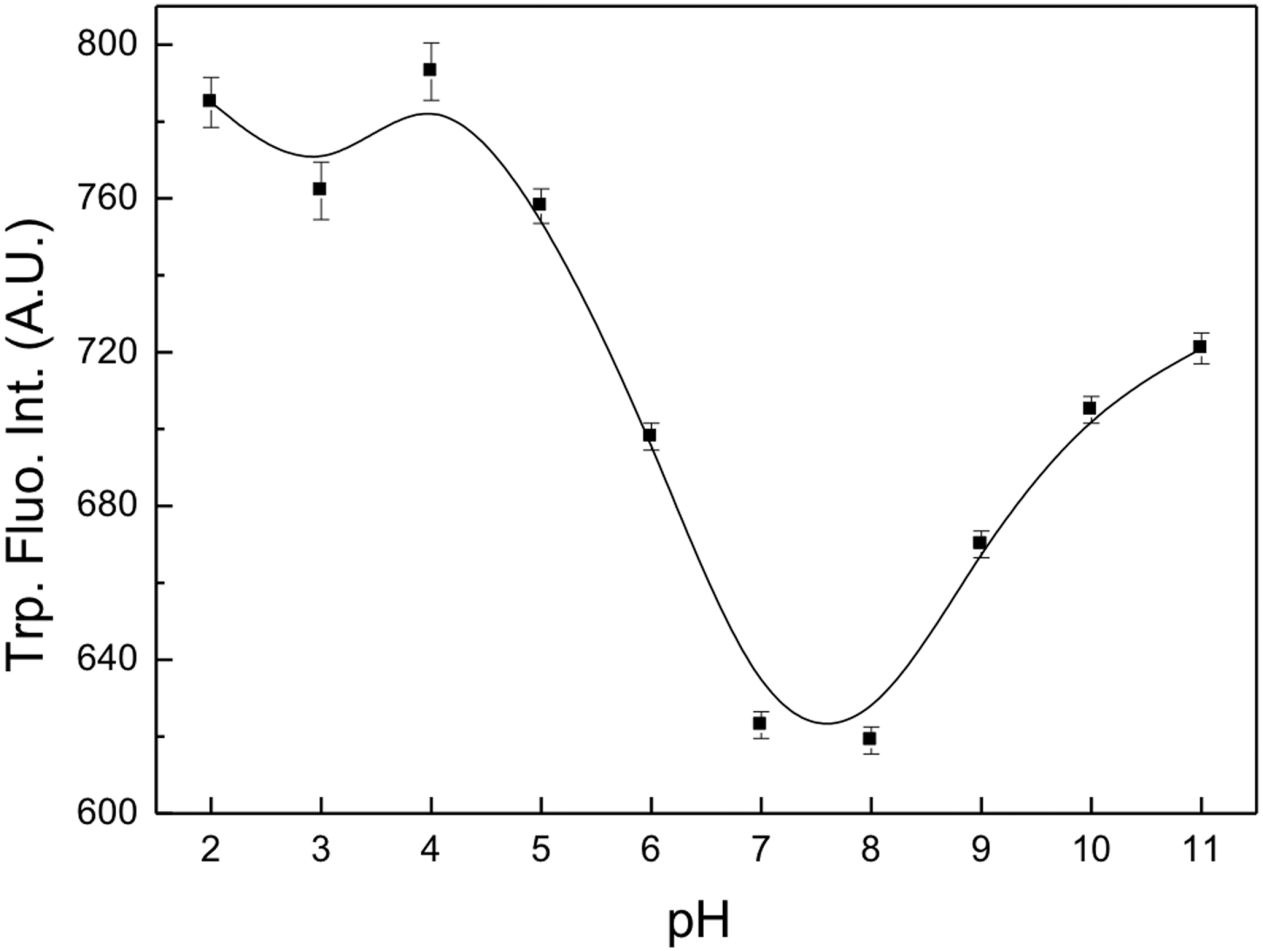
Titration of the intrinsic tryptophan fluorescence of sll0067. The binding of GSH to the protein was studied by quenching tryptophan fluorescence at various pH values. The GSH binds to the protein at physiological pH while at other pH values, it does not interact with the protein.

The binding of GSH to sll0067 at various pH values was studied using fluorescence spectroscopy. Fig 4 shows the decrease in the intensity of tryptophan fluorescence upon interaction with GSH at pH 7.0 and 8.0. Below pH 7.0 and above pH 8.0, the intensity increases steeply, indicating that GSH cannot bind at other pHs.

## Discussion

GSTs are the major detoxification enzymes in virtually all organisms. Despite being structurally conserved, the functional divergence of GST is tremendous, indicating its inevitable importance. GSTs have been widely studied for their functional roles in multiple metabolic pathways and stress responses. Structurally, most of the GSTs have marginal conformational stability with respect to temperature and pH changes. Only a few GSTs are reported to have considerably high thermal stability and can tolerate pH values between 5 to 11 [34–37]. We hereby report a novel GST with exceptionally high stability over a wide pH range of 2–11. The biochemical features of sll0067 have also been characterized. The results are important because GSTs are also being used for a variety of practical applications, including protein engineering and understanding the long term storage of proteins.

**Fig 5:**
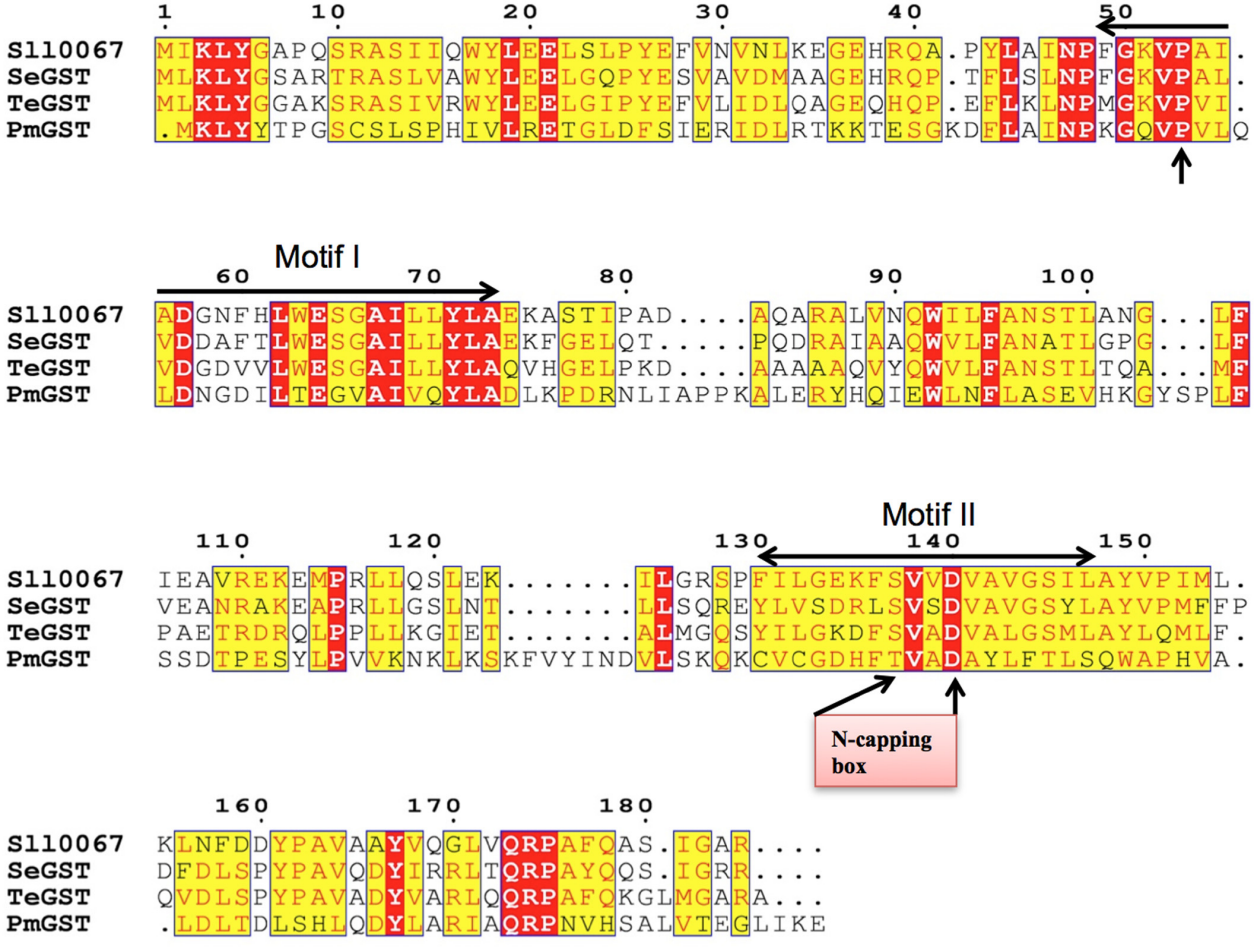
Multiple amino acid sequence alignment. sll0067 (Q55139), TeGST (NP_680998), SeGST (YP_171005) and PmGSTB1-1 (P15214) are aligned using ESpript 3.0 software, which utilizes the Clustal W algorithm. Similar residues are shown in yellow boxes; the red boxes represent identical amino acid residues while the amino acid residues with different properties have no boxes. The arrow represents conserved Pro-53. The motif I (49–73) and motif II (130–147) are shown with arrows.

Amino acid sequence similarity and substrate specificity suggests that sll0067 is a member of Chi-class GST (Fig 5). These GSTs particularly use isothocyanates as a substrate. Of them, sll0067 showed maximum catalytic activity with PITC. The kinetic parameters of sll0067 are presented in Table 1. The affinity (*K*m) of GSH was almost 5 fold less as compared to TeGST; however, it was comparable to that of SeGST [23] as it was within the normal range (below 1 mM), which is similar to that of many mammalian GSTs [38]. For PITC, the *K*m of sll0067 was 4 to 7 times higher than that of SeGST and TeGST and 3 to 5 times higher than that of mammalian GSTs [38], indicating that they have lower affinity than other cyanobacterial and mammalian GSTs. Therefore, sll0067 has a lower affinity for both GSH and PITC than its counterparts, TeGST, and SeGST as well as mammalian GSTs. The turnover of enzyme sll0067 (*K*cat) was almost similar to that of TeGST but three times less as compared to SeGST. The *K*cat/*K*m was 7 to 12 fold less than SeGST and TeGST, 4 to 5 fold less than GSTM1-1 and GSTP1-1 and 3 to 11 times more than GSTA1-1 and GSTM4-4 [23, 38]. The specificity constant *K*cat/*K*m value is a measure of the catalytic efficiency and represents the potential for catalysis at low substrate concentrations. The present findings, therefore, suggest that sll0067 has lower kinetic efficiency than most of the mammalian GSTs as well as TeGST and SeGST. The specific activity is higher for sll0067 as compared to the above described GSTs. Therefore, sll0067 along with the two Chi-class cyanobacterial GSTs, TeGST, and SeGST, are the first few examples of bacterial enzymes with high specific activity than the mammalian enzymes.

In addition, sll0067 has a broad substrate degradation capability, like most of the mammalian GSTs, compared to PmGST B1-1 and *E*. *coli* GST, which are typical prototype representatives for bacterial GSTs [21, 27] and show poor catalytic activity and low specificity towards standard GST substrates such as CDNB. The active site of any GST is composed of two subsites, the glutathione binding site (G-site) and the hydrophobic substrate-binding site (H-site) where the electrophiles bind. While the functional properties of the amino acid residues that make up the G-site of a GST are generally conserved among different classes, the residues forming the hydrophobic substrate-binding pocket vary considerably between different GSTs. Since the architecture of the H-site governs the substrate specificity of a particular GST, the variability in the H-site helps the GST family to catalyze reactions towards an exceedingly large number of structurally diverse substrates. The sequence alignment of sll0067 and PmGST B1-1 (Fig 5) showed less identity in the amino acid residues in the C-terminal region than N-terminus, clearly indicating the reason for the broad substrate range of sll0067.

**Fig 6:**
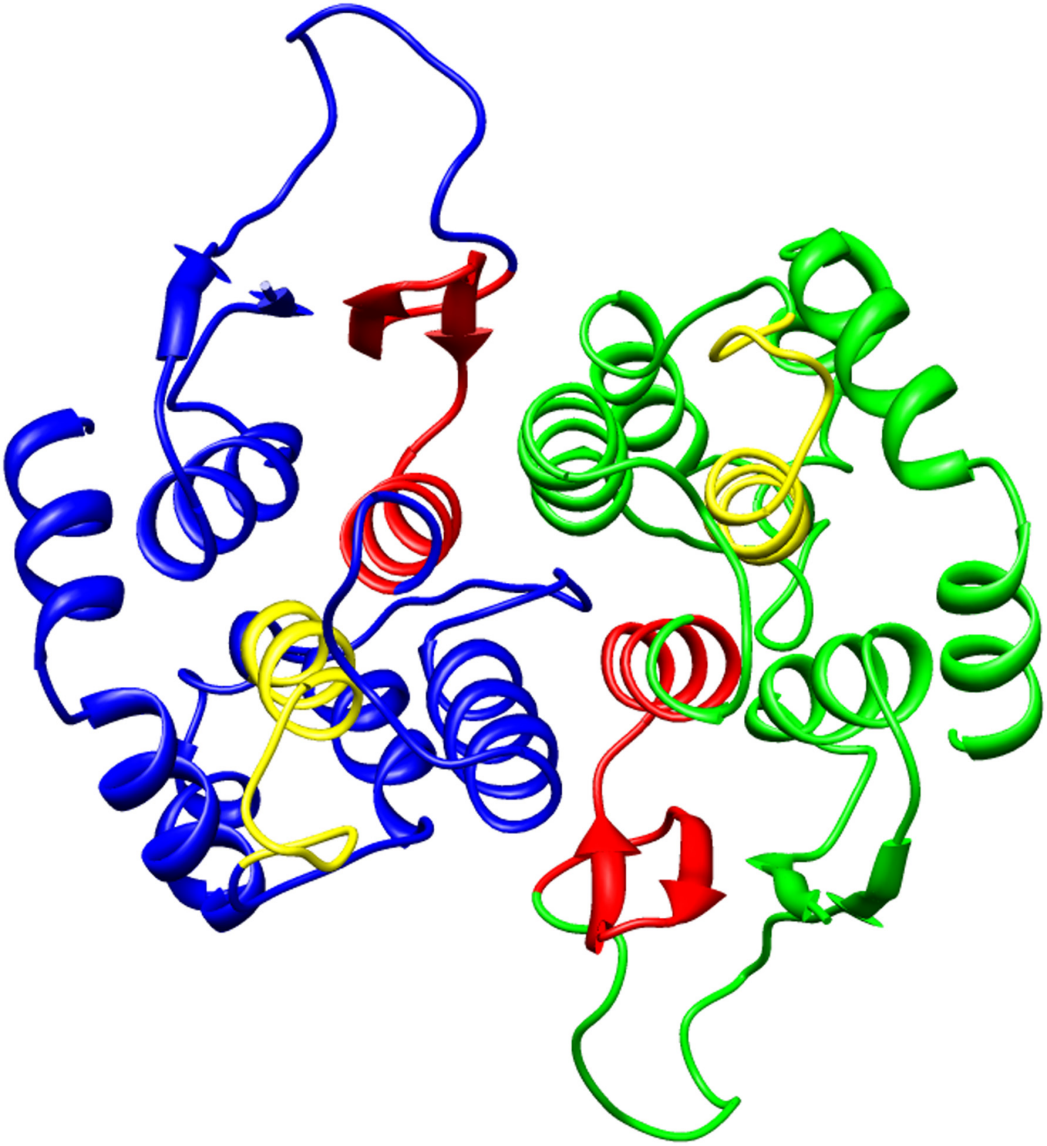
Molecular model of sll0067. The structure of a dimer is shown in this figure. The blue and green colors represent the two monomers. The red color shows motif I while the yellow color shows motif II. The 3D model was made using the Swiss model. The model was visualized with UCSF Chimera.

Chi-class GSTs are considerably smaller in size than most of the GSTs reported in bacteria or eukaryotes. In our quest to study the GSTs of cyanobacteria, we observed a novel feature of sll0067 of the model cyanobacterium- *Synechocystis* PCC 6803 and characterized it. The protein was expressed and purified to obtain a single band in SDS-PAGE. SEC revealed the dimeric nature of the protein, which is a common feature of most GSTs. The secondary structure prediction of sll0067 depicts that the N-terminal domain of about 75 residues adopts aα/β topology (typical thioredoxin- βαβαββα fold), whereas the C-terminal domain is all alpha-helical (Fig 6). This is similar to other Chi-class cyanobacterial sequences of similar lengths annotated as GSTs [23]. The N-terminal GSH-binding domain is highly conserved between the different GSTs while the C-terminal domain exhibits more structural variation, presumably to allow the recognition and binding of the structurally diverse range of electrophilic compounds that are known to be GST substrates [1, 6–9]. The amino acid sequence alignment showed that the Pro-53 is conserved in all cyanobacterial Chi-class GSTs viz. sll0067, TeGST and SeGST as well as in PmGST B1-1 (Fig 5). Crystal data from several GSTs indicate that this Pro-53 adopts the cis-configuration [10, 14, 39, 40], which is also present in homologous structures from the thioredoxin superfamily from which GSTs are thought to have evolved [25]. Pro-53 is located in β-turn that lines the base of the G-site and is thought to be important for the proper folding and maintenance of the G-site [41]. The two segments of amino acids, defined as GST motif I (residues 49–73 in TeGST and SeGST) and GST motif II (residues 130–147 in TeGST and 129–146 in SeGST respectively), are identified in sll0067 as well and are shown in the sequence alignment (Fig 5 and S1 Fig). This motif is also found in some non-GST proteins [42]. Within GST motif II, the local structural motifs, denoted as N-capping box and hydrophobic staple (Fig 5), are crucial for the folding of GSTs, which was previously shown for human GSTs [43–45] and bacterial GST [46] including PmGST B1-1 [37]. In PmGST B1-1, the N-capping box consists of a Thr-Xaa-Xaa-Asp motif, where a phenylalanine residue and an alanine residue constitute the hydrophobic staple motif. In the case of sll0067, TeGST, and SeGST, serine replaces threonine in the N-capping box and phenylalanine and valine residue constitute the hydrophobic staple motif except in SeGST in which leucine is present instead of phenylalanine. Aspartate-140 amino acid residue, which is a part of the N-terminal box, is thought to be involved in the stability and structural maintenance of GSTs [47]. The sequence alignment supports the idea that these residues were conserved during evolution because of their involvement in the folding and stability of cytosolic GSTs (8–10, 35). In addition, sll0067, like TeGST and SeGST, also lack cysteine residues at the N-terminus, which is involved in the catalysis and binding of GSH in PmGST B1-1. Concomitantly, due to less sequence similarity with PmGST B1-1, it is predicted to have a different evolutionary pathway for the cyanobacterial Chi-class GSTs, as interpreted earlier [23].

The structural stability of sll0067 at a wide pH range was studied using fluorescence and CD spectroscopy. Intrinsic fluorescence of the tryptophan residue has been extensively used as a spectral probe of tertiary structure that provides information about the solvent accessibility and hydrophobicity of tryptophan microenvironment [48]. sll0067 encodes three tryptophan residues in its amino acid sequence. The maximum emission wavelength of the tryptophan fluorescence for the recombinant sll0067 was observed at about 330 nm (S2 Fig). It is reported that the buried tryptophan residues in folded proteins show a fluorescence emission maximum at 330–335 nm whereas upon unfolding of proteins the emission maximum shifts to 350–355 nm. This observation suggests that the tryptophan residues in the native conformation of sll0067are not solvent accessible. No deviation in the tryptophan emission maxima after changing the pH of the protein solution from 2 to 11 suggests that the tertiary structure of the protein is not disturbed over a wide pH range (Fig 3A). Far-UV CD spectroscopy has been widely used to determine the secondary structure of the protein. The far-UV CD spectrum of sll0067 showed a typical αβ structure (S3 Fig). Minor loss of CD signal at 222 nm observed for the protein at low pH values could be due to the local unfolding of minimal secondary structures that were well intact from pH 6–11, indicating that there was no loss of secondary structure of the protein. Finally, the integrity of the quaternary structure was studied using SEC that showed no shift in the elution volume, establishing that the quaternary structure of the protein was intact. The compaction of protein at low pH occurs due to the deionization of polar amino acid residues present in the interior of the protein that leads to a decrease in electrostatic repulsions; this has been observed in many proteins [49, 50]. This further indicates that the unusual stability of sll0067 can be due to the attractive charge-charge interactions present in the protein.

The binding of GSH to the protein was investigated by monitoring the intrinsic tryptophan fluorescence of the enzyme. The substrate binding results in partial quenching of the fluorescence intensity due to direct interactions between the bound GSH and the indolefluorophore of the tryptophan [36, 51, 52]. We monitored the tryptophan fluorescence intensity of the sll0067 at various pH values. Partial quenching of the tryptophan fluorescence intensity was observed between pH 7.0 and 8.0, indicating the binding of GSH to the protein at these pH values. This result indicates that at non-physiological pH, the GSH molecule is not able to bind to the protein due to charge alterations and thus, the protein does not show functional activity at these pHs.

Refining our understanding of protein stability is essential for understanding protein structure, folding and function. The conformational stability of proteins depends on a delicate balance of a number of forces and interactions. Electrostatic interactions are well known to affect protein stability and can be both stabilizing and destabilizing. The electrostatic interactions in proteins may not be optimized for maximal stability due to functional restrains. Thus, studies on pH-dependent protein stability are not only useful in understanding the detailed balance of the forces and interactions in proteins but can also indicate the specific electrostatic interactions and functionally significant charged groups. The pH dependence of the stability of proteins is linked thermodynamically to the pKa values of titrable groups in the native and unfolded states. The degree of interactions between an ionizable residue and the rest of the protein in its native or denatured forms determines its titration properties. The pKa values depend, in turn, on charge-charge, charge-dipole, H-bonds and desolvation effects in the native and unfolded states. Most proteins unfold at low or high pHs (below 5 and above 10) because the folded protein has groups buried in non-ionized form that can ionize only after unfolding, particularly the His and Tyr residues that tend to cause unfolding at acid and alkaline pH, respectively. The high stability of sll0067 can be due to the constructive charge-charge and charge-dipole interactions that are important for maintaining the 3D structure of the protein. Further, we have attempted to solve the crystal structure of sll0067 so as to better understand the precise molecular basis of stability of this unique protein as well as elucidating the active site residues involved in the catalysis.

## Supporting Information

S1 Fig Secondary structure prediction for sll0067. The structural elements are indicated in the following letters- E, extended strand; H, helix. A dash indicates that structural data are not available or that the alignment algorithm has inserted a gap.
(DOCX)

S2 Fig Tryptophan emission spectrum of native sll0067. The tryptophan fluorescence emission spectrum was measured at 25°C in a Varian Cary Eclipse fluorescence spectrophotometer. The sample was excited at 280 nm in order to obtain the intrinsic tryptophan fluorescence spectrum. Both excitation and emission bandwidth was kept at 5 nm.
(DOCX)

S3 Fig Far-UV CD spectrum of native sll0067. The Far-UV CD spectrum was measured at an enzyme concentration of 2 μM with a 1 mm path length cell at 25°C in a Jasco J-815 spectropolarimeter.
(DOCX)
