# Supplementary material for: Characterization of a Highly pH Stable Chi-Class Glutathione S-Transferase from Synechocystis PCC 6803
Source: PLoS One. 2015 May 12;10(5):e0126811. doi: 10.1371/journal.pone.0126811 (PMC4429112; doi:10.1371/journal.pone.0126811)

**Figure S1. Secondary structure prediction for sll0067.** The structural elements are indicated in the following letters- E, extended strand; H, helix. A dash indicates that structural data are not available or that the alignment algorithm has inserted a gap.


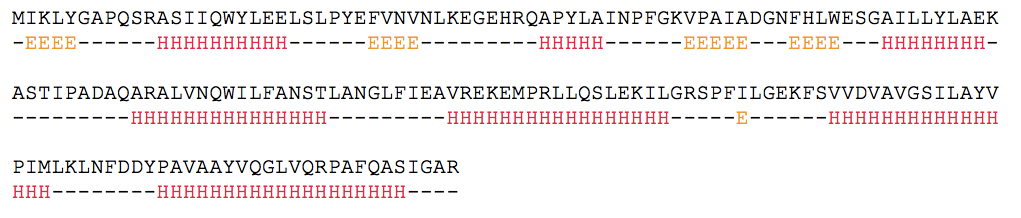

Supplement: S1 Fig — The structural elements are indicated in the following letters- E, extended strand; H, helix. A dash indicates that structural data are not available or that the alignment algorithm has inserted a gap. (DOCX) [file pone.0126811.s001.docx]
