# Supplementary material for: Characterization of a Highly pH Stable Chi-Class Glutathione S-Transferase from Synechocystis PCC 6803
Source: PLoS One. 2015 May 12;10(5):e0126811. doi: 10.1371/journal.pone.0126811 (PMC4429112; doi:10.1371/journal.pone.0126811)

**Figure S2. Tryptophan emission spectrum of native sll0067.** The tryptophan fluorescence emission spectrum was measured at 25 °C in a Varian Cary eclipse fluorescence spectrophotometer. The sample was excited at 280 nm in order to obtain the intrinsic tryptophan fluorescence spectrum. Both excitation and emission bandwidth was kept at 5 nm.


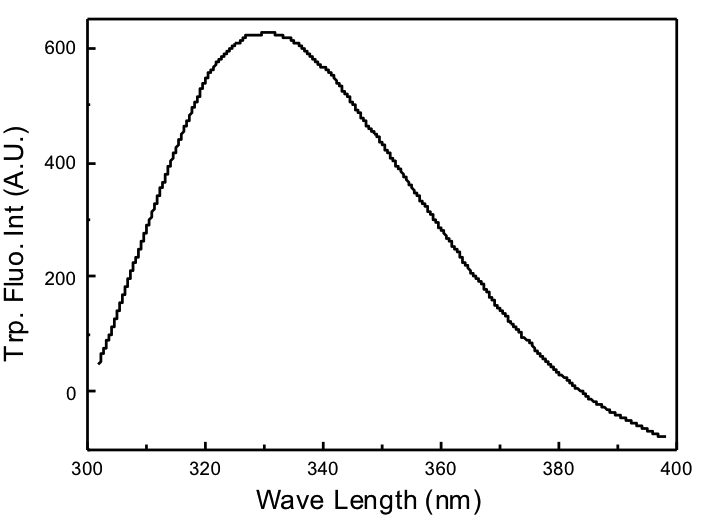

Supplement: S2 Fig — The tryptophan fluorescence emission spectrum was measured at 25°C in a Varian Cary Eclipse fluorescence spectrophotometer. The sample was excited at 280 nm in order to obtain the intrinsic tryptophan fluorescence spectrum. Both excitation and emission bandwidth was kept at 5 nm. (DOCX) [file pone.0126811.s002.docx]
