# Supplementary material for: Characterization of a Highly pH Stable Chi-Class Glutathione S-Transferase from Synechocystis PCC 6803
Source: PLoS One. 2015 May 12;10(5):e0126811. doi: 10.1371/journal.pone.0126811 (PMC4429112; doi:10.1371/journal.pone.0126811)

**Figure S3. Far-UV CD spectrum of native sll0067.** The Far-UV CD spectrum was measured at an enzyme concentration of 2 μM with a 1 mm path length cell at 25 °C in a Jasco J-815 spectropolarimeter.


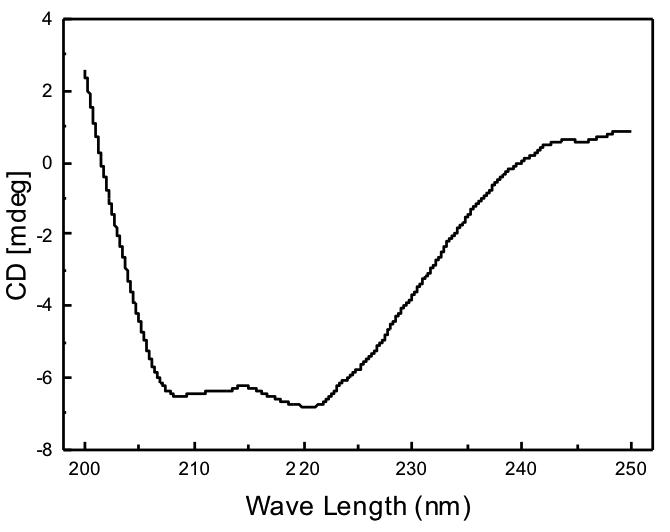

Supplement: S3 Fig — The Far-UV CD spectrum was measured at an enzyme concentration of 2 μM with a 1 mm path length cell at 25°C in a Jasco J-815 spectropolarimeter. (DOCX) [file pone.0126811.s003.docx]
